# Distinct Roles of SLC26A3 and CFTR in Surface pH Regulation and Bicarbonate Secretion in Human Intestinal Epithelium

**DOI:** 10.1111/apha.70157

**Published:** 2026-01-07

**Authors:** Mahdi Amiri, Azam Salari, Ursula Seidler

**Affiliations:** ^1^ Department of Gastroenterology Hannover Medical School Hannover Germany

**Keywords:** bicarbonate secretion, CFTR, cystic fibrosis, intestinal organoids, SLC26A3, surface pH

## Abstract

**Background and Aims:**

Colonic bicarbonate secretion is mediated by the chloride/bicarbonate exchanger SLC26A3 and the cystic fibrosis transmembrane conductance regulator (CFTR). Dysfunction of either causes luminal acidosis, altered mucus properties, and inflammation. While physical and functional interactions have been demonstrated in heterologous systems, their relationship in native epithelium is not fully established. We investigated the distinct roles of SLC26A3 and CFTR using human intestinal organoids with inducible SLC26A3 overexpression.

**Methods:**

Human colonic and rectal organoids from healthy controls and cystic fibrosis patients with F508del mutations were studied in the proliferative state with high endogenous CFTR expression and inducible SLC26A3 overexpression. Real‐time surface pH measurements, electrophysiological analysis, forskolin‐induced swelling assays, and confocal microscopy were employed.

**Results:**

Steady‐state surface pH was lower in CF versus healthy organoids (7.23 ± 0.03 vs. 7.34 ± 0.03). SLC26A3 overexpression normalized surface pH in CF organoids and CFTR‐inhibited organoids, equalizing responses between genotypes. SLC26A3 overexpression corrected abnormal morphology and significantly improved intracellular MUC2 distribution in CF organoids. However, SLC26A3 did not restore fluid secretion in CF organoids or enhance CFTR‐mediated electrogenic anion secretion in Ussing chambers.

**Conclusions:**

SLC26A3 and CFTR perform distinct yet complementary functions. SLC26A3 dominates surface pH regulation and maintains bicarbonate efflux independently of CFTR, while CFTR drives agonist‐stimulated fluid secretion. SLC26A3's ability to restore pH homeostasis and normalize mucin intracellular distribution in CF organoids demonstrates its critical importance for maintaining colonic mucosal health.

## Summary

- SLC26A3 independently maintains colonic surface pH and can compensate for CFTR dysfunction in cystic fibrosis.
- SLC26A3 overexpression normalized abnormal mucin distribution in CF intestinal organoids.
- Despite restoring surface pH, SLC26A3 cannot rescue CFTR‐dependent fluid secretion defects.

## 1 Introduction

Mucosal bicarbonate secretion in the colon is essential for maintaining mucosal transport function and tissue homeostasis by regulating both intracellular and extracellular pH (reviewed in refs [1, 2, 3]). Two anion transporters mediate this process at the apical membrane of the colonic epithelium: the chloride/bicarbonate exchanger SLC26A3 (also known as down‐regulated in adenoma, DRA) and the cystic fibrosis transmembrane conductance regulator (CFTR) [4, 5].

The physiological importance of bicarbonate efflux in the colon becomes evident in disorders such as cystic fibrosis (CF) and congenital chloride diarrhea (CLD), which arise from CFTR and DRA deficiencies, respectively. CFTR dysfunction leads to colonic luminal acidosis in both mice [6] and humans [7], associated with increased mucus viscosity, defective goblet cell degranulation, and impaired mucus expansion [8, 9, 10]. However, studies in mouse and human mid‐distal colon also suggest that the high basal output rates for ${\mathrm{HCO}}_{3}^{–}$ into the lumen are largely CFTR‐independent, while the anion and fluid secretory rates are strongly compromised in the absence of a functional CFTR [11, 12, 13, 14]. Loss of DRA function in mice and humans results in luminal acidosis, systemic alkalosis, and severe fluid and electrolyte loss [12, 15, 16]. DRA‐knockout mice show altered mucin composition, reduced goblet cell theca, and lack of firm mucus layer in colon [8, 12]. These changes in the tissue pH microclimate and the compromised protective mucus barrier consequently disrupt host‐microbiome interactions and promote mucosal inflammation and dysbiosis in both humans and mice [6, 8, 17, 18].

Under physiological conditions, CFTR predominantly facilitates chloride efflux, with bicarbonate accounting for approximately 25% of its total anion conductance [19]. When intracellular chloride concentrations are low, the permeability ratio of CFTR shifts to favor bicarbonate over chloride [20], underscoring the context‐dependent nature of CFTR transport properties. DRA serves as the principal Cl^−^/${\mathrm{HCO}}_{3}^{–}$ antiporter on the luminal surface of colonic epithelial cells, mediating exchange of bicarbonate for chloride [21, 22]. The bicarbonate‐export capacity of DRA is proposed to be enhanced through its functional interaction with CFTR, where anion efflux by CFTR maintains the subapical chloride concentration required for efficient Cl^−^/${\mathrm{HCO}}_{3}^{–}$ exchange by DRA [23, 24]. This functional coupling is facilitated by a physical interaction between the STAS (Sulfate Transporter and Anti‐Sigma factor antagonist) domain of DRA and the R (Regulatory) domain of CFTR, promoted by phosphorylation of the R domain by protein kinase A in response to elevated intracellular cAMP [23]. In cells coexpressing the sodium/hydrogen exchanger NHE3, the key partner of DRA in electroneutral NaCl absorption, cAMP stimulation is described to shift DRA coupling from NHE3‐mediated absorption toward CFTR‐driven secretion [25, 26, 27].

Reciprocal modulation between DRA and CFTR, particularly under secretagogue stimulation, has been investigated in multiple in vivo and in vitro systems. In heterologous expression models lacking endogenous CFTR, cAMP‐dependent stimulation of DRA activity occurs only when CFTR is coexpressed [28, 29]. Similarly, in intestinal models with native expression of both DRA and CFTR, such as mouse duodenal mucosa, Caco‐2 cells, and human colonoid cultures, cAMP appears to enhance DRA function [25, 30], while cAMP does not affect the maximal transport rate of human or murine DRA in HEK cells or Xenopus oocytes [29, 31].

Some recent studies propose direct activation of DRA via synergistic, calcium‐dependent effects of cAMP and ATP [32] or through the guanylate cyclase‐C agonist linaclotide [33]. Alternatively, in vitro and in vivo data suggest that both in the presence and absence of CFTR expression in the colon, the cAMP or cGMP‐mediated inhibition of proton export via the Na^+^/H^+^ exchanger isoform 3 results in a DRA‐dependent increase in ${\mathrm{HCO}}_{3}^{–}$ output into the lumen [12, 13].

It is long known that directional chloride transport in epithelia is accomplished by an array of transporters in the apical and basolateral membranes (reviewed in refs [34, 35, 36]). This can result in the sustained translocation of chloride ions across the epithelium during stimulation of secretion without major changes in intracellular volume or chloride concentration, unless the basolateral Cl^−^ uptake mechanisms are inhibited [37]. In contrast, the cellular systems used for heterologous coexpression studies of CFTR and SLC26 family members do not provide the cellular context in which such a rapid import of anions from the extracellular space/basolateral side is possible. This will favor chloride‐recycling via CFTR and Cl^−^/anion exchanger coexpressed in these systems [28, 29]. In isolated epithelia or cell monolayers, the removal of Cl from the luminal bath may result in very low intracellular Cl^−^ concentrations during CFTR stimulation and favor WNK activation, which changes the ${\mathrm{HCO}}_{3}^{–}$ permeability of the CFTR channel and inhibits apical SLC26 anion exchangers [20, 38]. This will favor CFTR‐dependent, SLC26 member‐independent ${\mathrm{HCO}}_{3}^{–}$ secretion.

To avoid either of these confounders, flag‐tagged DRA was expressed in a doxycycline‐inducible fashion in proliferating, nondifferentiated human colonic and rectal organoids from healthy (HL) individuals, which express high endogenous levels of CFTR as well as the basolateral Cl^−^ and ${\mathrm{HCO}}_{3}^{–}$ uptake transporters but low endogenous DRA levels [39, 40]. Rectal organoids from patients with F508del homozygous mutations (CF organoids) and no residual CFTR function as tested in intestinal current measurements techniques in freshly excised biopsies [41], were also studied. These organoids were studied in two‐dimensional monolayers to study surface pH‐maintenance and apical alkalinization rates by a recently adapted method originally developed by Saint Criq et al. [42], and electrophysiologically in Ussing chambers to study the effect of DRA on CFTR‐mediated electrogenic anion secretion. Three‐dimensional HL and CF organoids with and without DRA overexpression were also studied using forskolin‐induced swelling assays to assess potential interactions of CFTR and DRA on fluid secretion and mucus properties.

## 2 Results

### 2.1 Dynamic Surface pH Regulation in Human Rectal Organoid Monolayers

Long‐term surface pH measurements under homeostatic conditions (37°C, 5% CO_2_) provide an effective approach for real‐time monitoring of epithelial acid–base transporter activity at the apical surface [42]. Using this method, we previously demonstrated that differentiated Caco‐2 monolayers dynamically regulate their surface pH in accordance with DRA expression, with Krebs‐Ringer Bicarbonate (KRB) solutions initially added to the apical surface gradually reaching steady‐state values of pH 7.1 in wild‐type cells and pH 6.35 in DRA‐knockout cells [22]. However, since initially added KRB occasionally contains higher bicarbonate concentrations than what is later established at steady state, and the equilibration takes several hours, this approach does not allow direct measurement of bicarbonate secretion by the mucosal cells.

To address real‐time bicarbonate secretion in CFTR‐expressing human rectal organoid monolayers, we used two bicarbonate‐free solutions in addition to KBR. One was unbuffered isotonic NaCl, which allows direct measurement of bicarbonate secretion by the epithelium as previously demonstrated in in vivo assays using anesthetized murine models [27]. However, under 5% CO_2_ conditions, unbuffered NaCl is acidified to approximately pH 5. To exclude potential stimulation of mucosal bicarbonate secretion in response to low extracellular pH, we also used Tris–NaCl (8.6 mM Tris, 150 mM NaCl), which neutralizes CO_2_‐induced acidification to pH 7 while maintaining minimal buffering capacity against pH changes caused by cellular bicarbonate transport (Figure S1).

**FIGURE 1:**
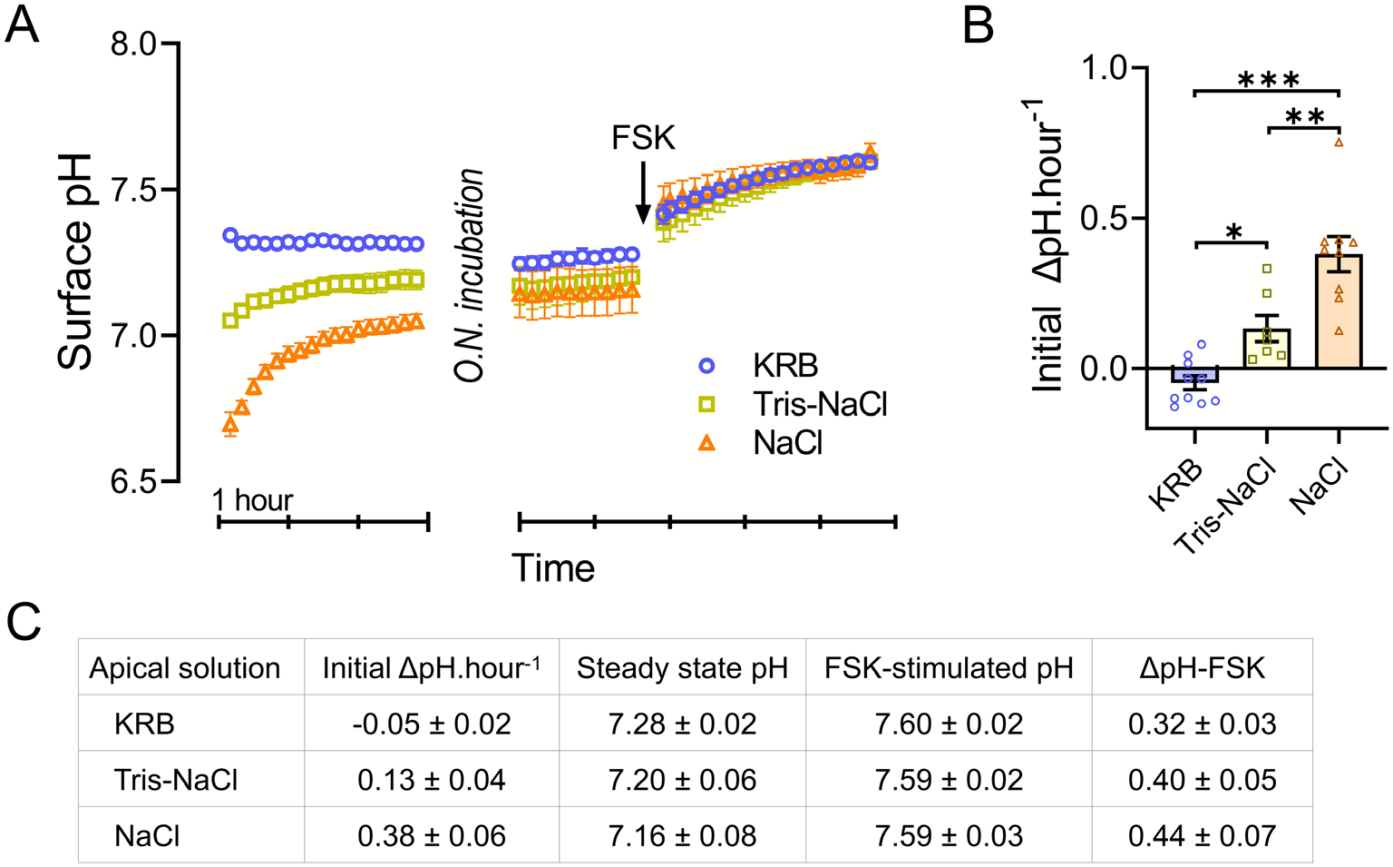
Establishment of stable apical surface pH in rectal organoid monolayers. (A) The apical surface pH of rectal organoid monolayers derived from a healthy donor (HL2) was measured using three different apical solutions: Unbuffered isotonic NaCl (orange triangles), isotonic NaCl containing 8.6 mM Tris (Tris–NaCl, yellow squares), and Krebs‐Ringer bicarbonate buffer (KRB, blue circles). The addition of 8.6 mM Tris maintains the NaCl solution at pH 7 in 5% CO_2_. The starting surface pH, which is imposed by the experimental apical solution, is actively regulated by the cells and reaches a stable steady‐state pH after overnight incubation. The rate of pH change in the first few hours is proportional to the starting pH deviation from the steady‐state pH. The steady‐state pH values for all conditions are in a comparable range, yet slightly higher for the KRB samples. Basolateral forskolin treatment enhances apical bicarbonate secretion and surface alkalinization similarly across all conditions; *n* ≥ 8. (B) A graph depicting the initial ΔpH per hour (ΔpH hour^−1^) calculated from the nominal maximum rate of pH changes during the initial phase of the experiment. (C) Values from (A) and (B), presented as Mean ± SEM. ANOVA, *p ≤ 0.05, **p ≤ 0.01, ***p ≤ 0.001.

This experimental approach enabled us to measure bicarbonate secretion in response to different starting apical pH conditions (KRB 7.4, Tris–NaCl ~7.0, and unbuffered NaCl ~6.5). The initial rate of pH change was highest in unbuffered NaCl, followed by Tris–NaCl, while KRB showed a slight negative slope (Figure 1A–C). The epithelial cells demonstrated a remarkable ability to establish a constant steady‐state surface pH after several hours (Figure 1C), regardless of the initial conditions. After reaching the steady state, the addition of forskolin to the lower chamber stimulated a similar bicarbonate secretion response across all conditions, identified as an immediate rise in the surface pH which came to a plateau after approximately 2 h (Figure 1A,C).

These results demonstrate that the robust capacity of intestinal epithelial cells to maintain surface pH homeostasis can be characterized by monitoring real‐time changes in the surface pH under physiological conditions in vitro. Using the bicarbonate‐free Tris–NaCl solution with a neutral initial pH allows for the detection of bicarbonate secretion rates by mucosal cells while excluding the probability of acid‐induced stimulation of bicarbonate secretion.

### 2.2 Distinct Roles of DRA and CFTR in Regulation of Surface pH in the Large Intestine

Previous studies have established distinctive distribution patterns of DRA and CFTR along the crypt/surface axis of human colonic epithelium. SLC26A3 exhibits highest expression at the epithelial surface, with gradually decreasing levels toward the base of the crypts [26, 40, 43, 44]. In contrast, CFTR shows maximal expression at the crypt base with moderate expression in the crypt neck region [44, 45, 46, 47]. While direct co‐staining of DRA and CFTR in human colon samples has been challenging due to antibody limitations, independent staining patterns suggest their expression may overlap in the crypt neck region ([48]; Atlas of Intestinal Transport https://jrturnerlab.com/database‐viewer/atlas‐of‐intestinal‐transport/ [49]). Emerging single cell sequencing data also confirm a population of the epithelial cells that coexpress *SLC26A3* and *CFTR* to substantial levels [25, 50].

**FIGURE 2:**
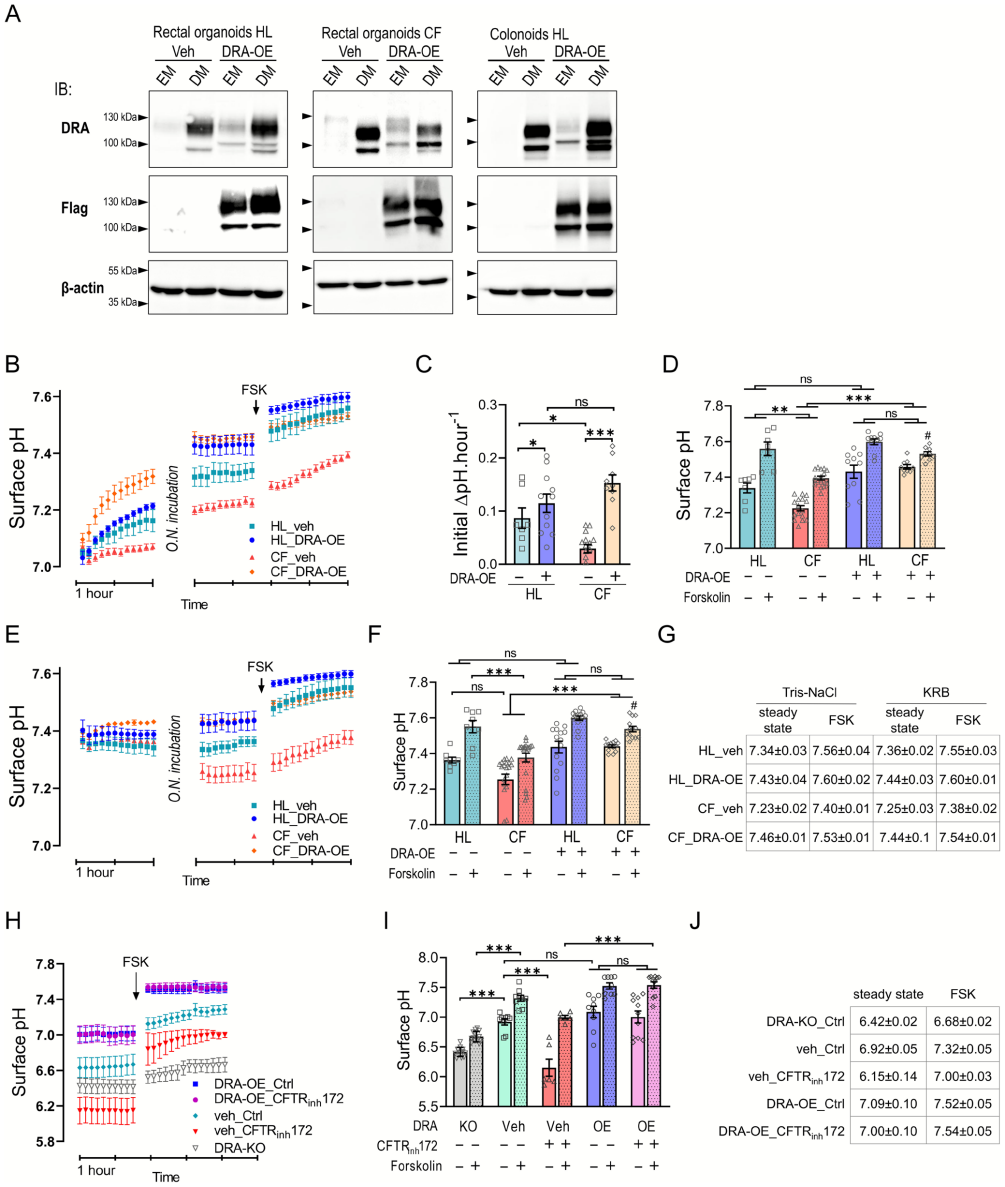
CFTR deficiency attenuates apical surface pH in rectal organoid monolayers, which is compensated by DRA overexpression. (A) Immunoblot data showing the expression of flag‐tagged DRA protein in healthy (HL) and cystic fibrosis (CF) rectal organoids and healthy colonoid cultures. (B–D) The apical surface pH of rectal organoid monolayers established from a healthy (HL) and a cystic fibrosis (CF) donor was measured using Tris–NaCl solution. Cultures were transduced with vehicle control (veh) or DRA constructs. CFTR deficiency substantially reduced the rate of surface alkalinization in the initial phase of the assay (C) and resulted in a significantly lower steady‐state surface pH after overnight incubation and a protracted response to forskolin (FSK) compared to the healthy control (D). DRA overexpression (DRA‐OE), on the other hand, boosted the initial alkalinization rate in both HL and CF cultures in a similar manner, resulting in a comparable steady‐state surface pH for these cultures which was not different from that of the HL vehicle control cultures. (E, F) Using KRB for apical surface measurement elevated the steady‐state surface pH of the CF vehicle control cultures and minimized their differences with other conditions; the overall trend, however, recapitulated the Tris–NaCl experiment set. The increase in surface pH in response to FSK defined at 2 h post‐treatment was statistically significant for all samples in B and E (paired *t*‐test, *p* < 0.001). (G) Steady‐state and FSK‐stimulated surface pH values for different cultures measured in either Tris–NaCl or KRB starting solution were highly comparable. (H) Measurement of the apical surface pH in colonoid cultures derived from a healthy donor using KRB solution. Inhibition of CFTR by 10 μM CFTR_inh_172 significantly reduced surface pH with minor effect on the response to forskolin stimulation compared to control (Ctrl) cultures. Inhibition by CFTR_inh_172 did not alter the steady‐state surface pH in colonoids overexpressing DRA (DRA‐OE). DRA‐knockout (DRA‐KO) colonoids exhibited a slightly reduced surface pH and a diminished response to forskolin compared with control cultures. (I) Bar graph illustration and (J) values for the steady‐state and forskolin‐stimulated apical surface pH values for each condition in colonoids. The increase in surface pH in response to FSK defined at 2 h post‐treatment was statistically significant for all samples in H (paired *t*‐test, *p* < 0.01). Data from healthy colonoids (HL1), healthy rectal organoids (HL2), and CF rectal organoids (CF17). Mea*n* ± SEM; *n* ≥ 6; one‐way ANOVA or Student's *t*‐test; ns (not significant) *p* > 0.05, *p ≤ 0.05, **p ≤ 0.01, ***p ≤ 0.001; ^#^ *p* ≤ 0.01 Student's *t*‐test versus HL_DRA‐OE plus FSK.

To elucidate the distinct physiological functions of DRA and CFTR in human large intestine, we utilized rectal organoids derived from healthy (HL) and cystic fibrosis (CF; ΔF508 homozygous) donors. These organoids were either transduced as vehicle controls (veh), expressing minute baseline DRA levels, or engineered to overexpress flag‐tagged DRA (DRA‐OE) under a Tet‐On promoter while maintaining endogenous CFTR expression. This system allowed us to selectively control DRA expression without compromising the expression of CFTR and of the basolateral anion uptake mechanisms in nondifferentiated organoids. We also used rectal organoids established from a cystic fibrosis patient with a homozygous Δ508F mutation to model CFTR deficiency. DRA expression in these models was characterized by immunoblotting (Figure 2A).

Surface pH regulation dynamics were investigated using fluorescent dyes diluted in both Tris–NaCl and KRB apical solutions. In Tris–NaCl conditions, CF control organoids exhibited significantly reduced initial bicarbonate output and surface alkalinization rates compared to HL counterparts (Figure 2B,C). Notably, DRA‐OE markedly increased the initial rate of surface alkalinization in both HL and CF samples compared to their respective controls, equalizing the responses between HL and CF organoids (Figure 2B,C). Upon establishing the steady state conditions, control CF cultures showed a significantly lower surface pH compared to HL cultures (Figure 2B,D). However, the CF_DRA‐OE cultures also demonstrated increased alkalinization rates and reached a surface pH level comparable to the HL_DRA‐OE cultures (Figure 2B,D). Forskolin stimulation induced surface alkalinization across all cultures, though CF_veh samples showed significantly diminished alkalinization rates, and steady state surface pH, compared to other conditions. The CF_DRA‐OE samples had slightly reduced forskolin responses compared to HL_DRA‐OE, aligning more closely with HL_veh responses (Figure 2B). These differences, however, did not reach statistical significance (Figure 2D).

Parallel experiments using KRB buffer as the apical solution yielded remarkably similar results, despite the inability to monitor initial alkalinization rates due to pre‐existing bicarbonate in the solution (Figure 2E,F). Notably, steady‐state and forskolin‐stimulated surface pH values were strikingly consistent between Tris–NaCl and KRB conditions across all experimental groups (Figure 2G).

To further investigate the function of DRA and CFTR in intestinal bicarbonate transport, we extended our analysis to human colonoid cultures (Figure 2H–J). Similar to the rectal organoid models, vehicle control and DRA‐OE colonoid monolayer were used for this purpose. To assess surface pH in the complete absence of the minor background DRA expression in the nondifferentiated colonoids, we also generated DRA‐knockout (DRA‐KO) colonoids using CRISPR/Cas9 gene editing.

Control colonoid cultures maintained a steady‐state surface pH of 6.92 ± 0.05, markedly lower than rectal organoids (7.34 ± 0.03) but comparable to our previously characterized Caco‐2 monolayers (7.06 ± 0.03; ref. [22]). Given this, we focused our analysis using KRB buffer without incorporating Tris–NaCl conditions. We used 10 μM CFTR_inh_172 to inhibit CFTR in human colonoids. CFTR inhibition resulted in substantial surface acidification (ΔpH ~0.8) in control colonoids. This decline in surface pH is more pronounced compared to CF rectal organoids (ΔpH ~0.11). Despite this acidification, forskolin‐stimulated bicarbonate secretion remained largely intact in CFTR‐inhibited colonoids, with only a modest, non‐significant reduction in alkalinization rate.

DRA‐OE maintained control‐like steady‐state surface pH (7.09 ± 0.10) even with CFTR inhibition (7.00 ± 0.10). Notably, DRA‐OE cultures exhibited rapid surface alkalinization upon forskolin stimulation, achieving maximal response at the first measurement post‐stimulation regardless of CFTR status. This enhanced bicarbonate secretion remained stable throughout the assay period (Figure 2H). Under CFTR inhibition, the forskolin‐induced pH elevation was significantly higher in DRA‐OE cultures compared to vehicle controls (Figure 2I,J).

Despite the minimal baseline DRA expression in nondifferentiated colonoids, DRA‐KO cultures displayed a modest but statistically significant reduction in steady‐state surface pH (6.42 ± 0.02) compared to vehicle control colonoids (6.92 ± 0.05; *p* ≤ 0.001), confirming that even this low level of endogenous DRA contributes to basal bicarbonate secretion (Figure 2H,J). Furthermore, DRA‐KO cultures exhibited a significantly attenuated response to forskolin stimulation compared to control cultures (6.68 ± 0.02 vs. 7.32 ± 0.05; *p* ≤ 0.001), indicating that DRA activity is required for maximal cAMP‐stimulated bicarbonate secretion in colonoids (Figure 2H–J).

The distinct steady‐state pH profiles and varying responses to CFTR dysfunction between colonoid and rectal organoid cultures suggest that the machinery that regulates surface pH in association with CFTR and DRA may differ between these models. These findings were confirmed using organoid cultures from additional donors (Figure S2). Nevertheless, both models demonstrate that in the lack of DRA function, CFTR deficiency substantially compromises epithelial bicarbonate secretion capacity, resulting in reduced steady‐state surface pH under physiological conditions. Enhanced DRA function can effectively compensate for reduced bicarbonate output caused by CFTR deficiency, promoting surface alkalinization to levels comparable with healthy controls at steady state and restoring forskolin responsiveness.

### 2.3 DRA Expression Slightly Increased Initial Swelling Rates in Healthy but Not CF Rectal Organoids

Anion output by CFTR plays an established role in regulating fluid secretion in the intestinal epithelium. Therefore, we investigated whether DRA coexpression with CFTR influences CFTR‐mediated anion output and epithelial fluid secretion.

A characteristic morphological feature of CFTR deficiency in intestinal organoids is shrunken lumina with eccentric shapes compared to non‐CF controls. This alteration primarily results from impaired ion secretion into organoid lumina due to CFTR dysfunction, despite continued ion absorption by other apical transporters [51].

**FIGURE 3:**
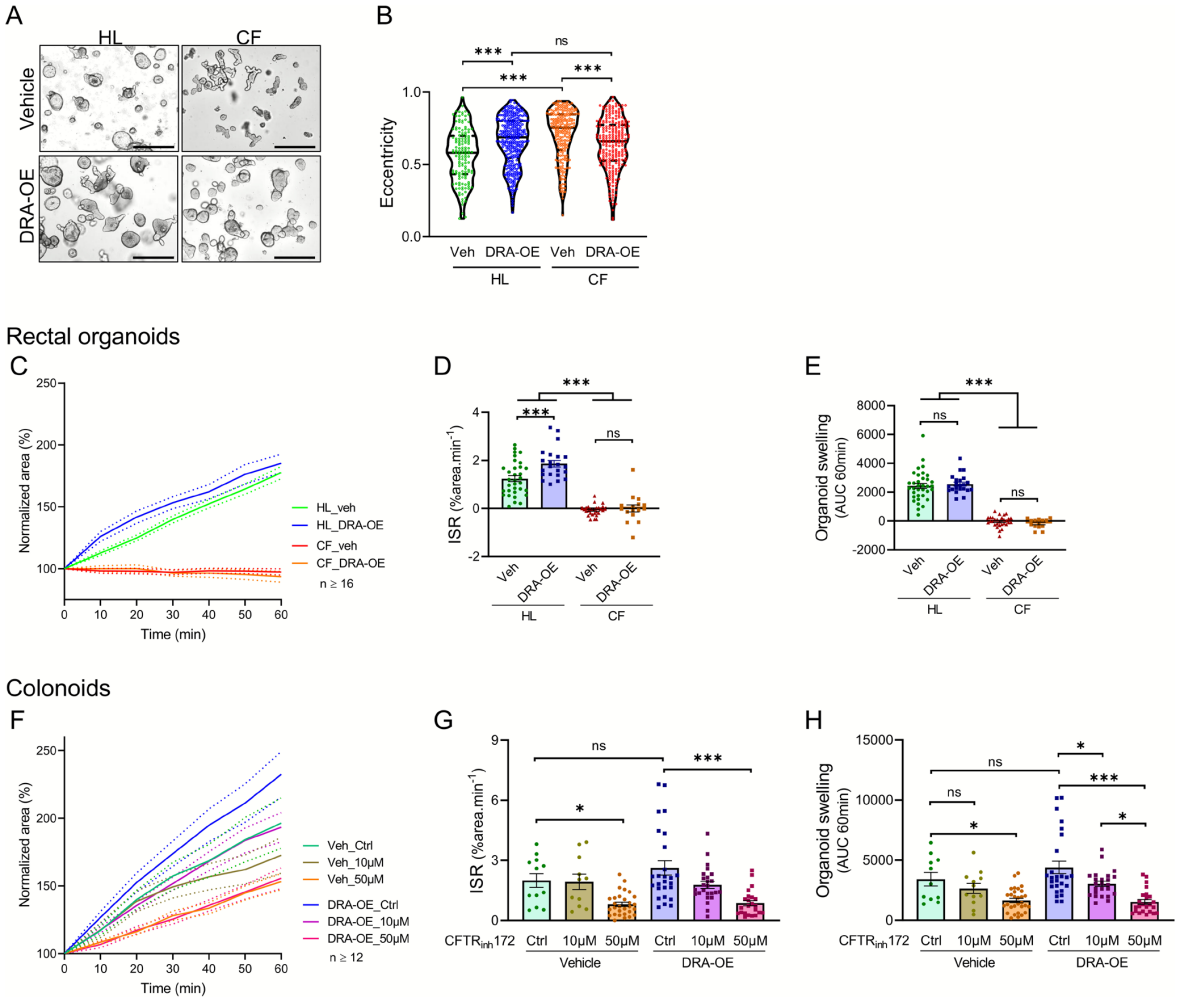
DRA has modest effects on CFTR‐associated epithelial fluid secretion. (A) Representative bright‐field images of 3D rectal organoids from healthy (HL) and cystic fibrosis (CF) donors under control conditions, showing morphological differences between CF and HL organoids. Scale bar represents 500 μm. (B) Quantitative analysis of organoid eccentricity in cross‐section for different experimental groups. Each data point represent one organoid structure. CF organoids exhibited significantly higher eccentricity values compared to HL organoids. DRA‐OE reduced eccentricity in CF organoids but increased it in HL organoids, ultimately diminishing differences between CF and HL organoids. (C) Forskolin‐induced swelling (FIS) assay results for rectal organoids. Unlike HL rectal organoids, CF organoids exhibited no response to forskolin stimulation regardless of DRA expression. (D, E) Quantitative analysis of initial swelling rate (ISR) and cumulative swelling (area under curve, AUC) for rectal organoids. (F) FIS assay results for colonoid cultures with and without CFTR inhibition using CFTR_inh_172 (10 or 50 μM). (G, H) Quantitative analysis of ISR and cumulative swelling for colonoid cultures. DRA‐OE appeared to improve both parameters compared to vehicle controls, though differences did not reach statistical significance. CFTR inhibition at 50 μM significantly reduced both ISR and overall swelling. Data from healthy colonoids (HL1), healthy rectal organoids (HL2), and CF rectal organoids (CF17). Mean ± SEM; one‐way ANOVA: Ns (not significant) *p* > 0.05, *p ≤ 0.05, **p ≤ 0.01, ***p ≤ 0.001.

As shown in Figure 3A, our bright field images confirmed morphological differences between CF and HL three‐dimensional rectal organoids under control conditions. Interestingly, upon DRA‐OE we observed improved roundness caused by increased luminal volume in CF organoids compared to vehicle CF controls. We quantified these phenotypic differences among different organoid models using eccentricity in cross‐section of the organoids, where values closer to zero indicate more circular structures and values approaching one represent increasingly elongated forms.

The data confirmed that CF organoids exhibited significantly higher eccentricity values compared to HL organoids. Remarkably, DRA‐OE resulted in reduced eccentricity in CF organoids compared to vehicle CF controls. Conversely, DRA‐OE in HL organoids increased eccentricity compared to vehicle HL controls. These opposing effects ultimately diminished differences in eccentricity between DRA‐OE CF and DRA‐OE HL organoids, suggesting normalization of CF organoid morphology through DRA‐OE under steady‐state conditions (Figure 3B).

We used forskolin‐induced swelling (FIS) assay in these models to investigate agonist‐stimulated fluid secretion in relation to CFTR and DRA functionality (Figure 3C–H; Figure S3). In HL rectal organoids, forskolin stimulation induced progressive expansion, with the normalized area increasing to approximately 180% of baseline over 60 min (Figure 3C). DRA‐OE in HL organoids enhanced the initial swelling rate (ISR) by approximately 50% (1.86 ± 0.14 vs. 1.24 ± 0.12% min^−1^; Figure 3D), though the cumulative swelling after 60 min showed no significant difference compared to controls (2553 ± 139 vs. 2426 ± 177 AUC; Figure 3E). In stark contrast, CF rectal organoids exhibited no response to forskolin stimulation throughout the observation period. DRA‐OE in CF organoids did not influence this swelling defect (Figure 3C–E).

To further investigate these dynamics in the colonic epithelium, we subjected colonoid cultures to similar experimental conditions, with the additional feature of pharmacological CFTR inhibition using CFTR_inh_172 at 10 or 50 μM concentrations. Control colonoids demonstrated robust forskolin‐induced swelling, achieving approximately 200% normalized area expansion after 60 min (Figure 3F). DRA‐OE appeared to increase both the ISR (2.63 ± 0.36 vs. 2.00 ± 0.34% min^−1^; Figure 3G) and cumulative swelling (4398 ± 520 vs. 3421 ± 561 AUC; Figure 3H) compared to vehicle controls, though these differences did not reach statistical significance. At 10 μM CFTR_inh_172, vehicle control and DRA‐OE colonoids showed only modest reduction in swelling parameters, which was insignificant for the vehicle control and statistically significant for the DRA‐OE colonoids. Both models exhibited a significant decrease in ISR and overall swelling at the 50 μM inhibitor concentration (Figure 3G,H).

These findings suggest that coexpression of DRA has minimal effects on the forskolin‐induced anion output by CFTR and its consequent fluid secretion in both colonic and rectal epithelium. CF rectal organoids exhibited complete abolishment of forskolin‐induced swelling, while 10 μM CFTR_inh_172 had a negligible effect on colonoid swelling and even at an elevated concentration of 50 μM produced only partial inhibition. A reduced inhibitory effect of CFTR_inh_ on intestinal fluid secretion or short circuit current response in isolated mucosae (as opposed to monolayer cultures) has been frequently observed before and may be caused by diffusion barriers for the substance. A similar situation may exist in three dimensional organoids embedded in extracellular matrix.

### 2.4 DRA Does Not Increase CFTR‐Mediated Anion Secretion in Human Intestinal Epithelia

**FIGURE 4:**
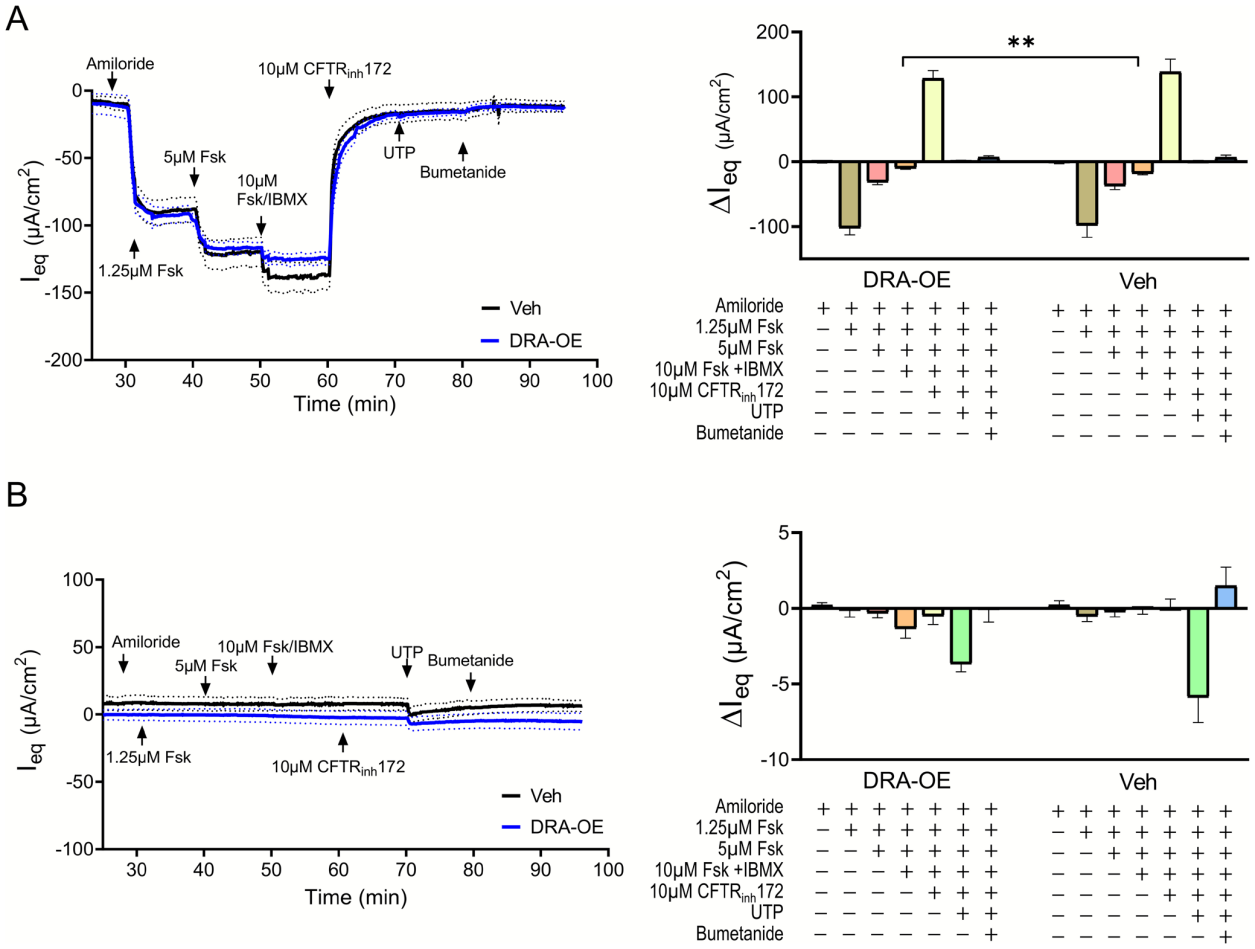
DRA expression does not increase CFTR‐mediated anion secretion in human rectal organoids. Ussing chamber electrophysiological recordings for HL (A) and CF (B) rectal organoids as vehicle control (veh) or DRA overexpressing (DRA‐OE) showing equivalent short‐circuit current (I_eq_) responses to different pharmacological stimulants and inhibitors. Data from healthy rectal organoids (HL2) and CF rectal organoids (CF17). Mean ± SEM, *n* ≥ 5; Student's *t*‐test: **p ≤ 0.01.

We further assessed the functional interaction between DRA and CFTR in anion transport by electrophysiological measurements in Ussing chambers. HL rectal organoid monolayers showed robust responses to sequential addition of 1.25 and 5 μM forskolin (Figure 4A). Addition of 10 μM forskolin with IBMX produced only modest additional effects. DRA‐OE monolayers exhibited similar responses, with no significant enhancement of CFTR‐mediated currents compared to vehicle controls. Inhibition of CFTR with CFTR_inh_172 abolished forskolin‐induced currents across all conditions and eliminated differences between groups, confirming that the observed secretory responses were CFTR‐dependent. Subsequent addition of UTP and bumetanide produced minimal effects with no significant differences between conditions, suggesting negligible contributions from calcium‐activated chloride channels or other NKCC1‐dependent transport mechanisms after CFTR inhibition.

CF rectal organoids demonstrated severely impaired anion secretion responses to all forskolin concentrations tested, consistent with their CFTR dysfunction (Figure 4B). Notably, DRA‐OE in CF organoids did not restore or significantly improve the forskolin‐induced currents, indicating that enhanced DRA expression cannot influence defective CFTR anion transport.

These findings demonstrate that DRA expression does not enhance CFTR‐mediated anion secretion in human intestinal epithelia. The findings contrast with the robust DRA‐mediated CFTR activation previously reported in heterologous expression systems [23].

### 2.5 DRA Expression Normalizes Mucin Distribution in CF Rectal Organoids

CFTR dysfunction in mucoviscidosis and CFTR null mice is associated with goblet cell hyperplasia, defective mucin degranulation, and the presence of viscid mucus on the epithelial surface [9, 10, 52]. We employed immunofluorescence confocal microscopy to compare mucin secretion in our HL and CF rectal organoid models as vehicle control or with DRA‐OE.

**FIGURE 5:**
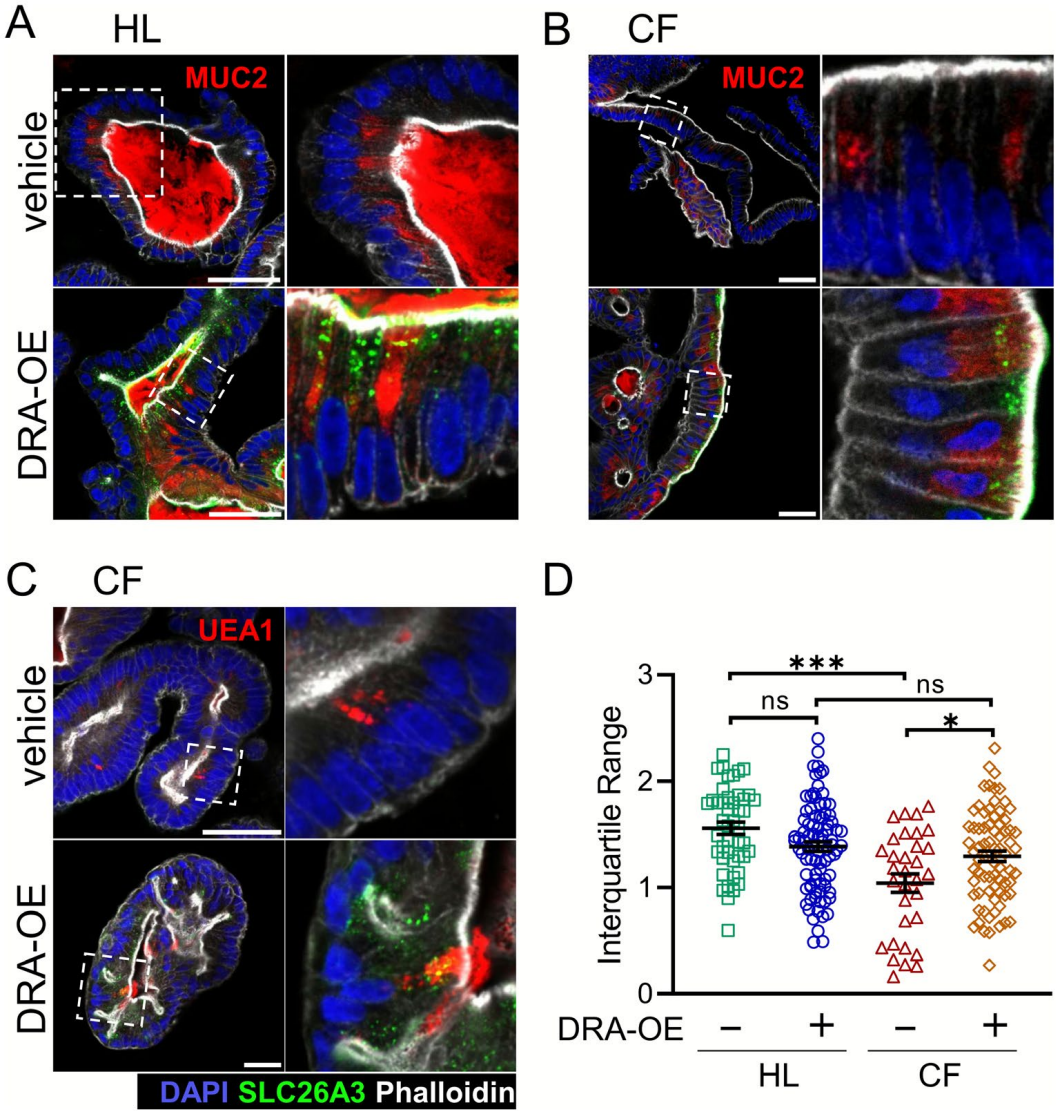
Dra expression normalizes intracellular mucin distribution in CF rectal organoids. (A–C) Representative confocal immunofluorescence images of rectal organoids stained for MUC2 or UEA1 lectin (red), DRA (green), F‐Actin with phalloidin (light gray), and nuclei with DAPI (blue). HL organoids showed broadly distributed mucin granules between nucleus and apical membrane, while CF organoids displayed altered distribution with larger, more densely packed MUC2‐positive granules and reduced abundance in the subapical compartment. DRA‐OE substantially improved mucin distribution in CF organoids. Scale bar = 50 μm. (D) Quantitative analysis of MUC2 signal distribution profiles along the goblet cell longitudinal axis. Interquartile range (Q3–Q1) values representing the spread of MUC2 signal distribution along the basal (0%) to apical (100%) membrane axis in individual goblet cells. Data from healthy rectal organoids (HL2) and CF rectal organoids (CF17). Each data point represents one cell. *n* ≥ 32, Mean ± SEM, ANOVA, ns = not significant, *p < 0.05, ***p < 0.001.

We used MUC2 antibody and UEA1 lectin staining to visualize intracellular mucin granules within the organoids (Figure 5). Due to the undifferentiated nature of our organoid cultures, goblet cells were relatively scarce compared to fully differentiated models, consistent with previous observations [40]. The presence of microvilli visualized by strong phalloidin signal at the apical side of these mucin‐producing cells further suggested their identity as primarily non‐canonical goblet cells [53].

In healthy rectal organoids, MUC2 immunostaining revealed mucin granules that were broadly distributed along the secretory pathway between the nucleus and apical membrane (Figure 5A). In contrast, CF organoids displayed markedly altered mucin distribution patterns characterized by larger, more densely packed MUC2‐positive granules that exhibited reduced abundance in the subapical compartment of the cells (Figure 5B).

Notably, DRA‐OE substantially improved mucin distribution in CF organoids, resulting in expanded MUC2 distribution patterns with enhanced presence at both subapical and apical cellular compartments. UEA1 staining yielded similar results (Figure 5C). Quantitative analysis of MUC2 signal distribution confirmed that DRA‐OE significantly influences intracellular mucin distribution in CF rectal organoids (Figure 5D).

## 3 Discussion

As detailed in the introduction, luminal alkalinization in the intestinal epithelium is predominantly mediated by the chloride/bicarbonate exchanger SLC26A3 (DRA) and the CFTR anion channel [1, 54]. While both transporters contribute to luminal alkalinization, their distinct distribution patterns along the crypt‐surface axis have complicated investigations of their functional interactions in native tissue. In the absence of colocalization data, individual immunohistological stainings and emerging sc‐RNA sequencing data suggest the existence of some epithelial cell populations coexpressing both transporters, providing a physiological basis for investigating their functional interplay [25, 49, 50].

Modeling this population of mucosal cells in human colonoids has been challenging in vitro due to the differential expression of both transporters during enterocyte differentiation [39, 40]. Previous studies used partially differentiated colonoid cultures, with lower CFTR compared to nondifferentiated and lower DRA expression compared to differentiated cultures, respectively [26, 50]. We developed human colonic and rectal organoid models that combine fully preserved endogenous CFTR expression with CRISPR/Cas9‐mediated DRA deletion or with inducible DRA overexpression. The nondifferentiated organoids that we use provide the full armamentarium for basolateral Cl^−^ and ${\mathrm{HCO}}_{3}^{–}$ uptake (NKCC1, NBCs, AE2) as well as for paracellular Na^+^ and water flux (Claudin 2) [40]. The experimental approach allowed us to study the mid colonic and rectal enteroids both as three‐dimensional organoids and as two‐dimensional monolayers, which permitted the study of CFTR and DRA function separately, and in conjunction. We used organoids from patients with homozygous F508del mutations and severely impaired CFTR function. DRA expression was manipulated by CRISPR‐mediated knockout. The system allowed us to test the hypothesis that a direct CFTR‐DRA interaction provides a higher anion efflux through either, or both, transporters, without favoring this very interaction by the lack of alternative anion uptake mechanisms.

Real‐time surface pH measurements in homeostatic conditions revealed differences in how DRA and CFTR contribute to epithelial bicarbonate secretion. We also observed regional differences in surface pH regulation, with rectal organoids maintaining a steady‐state surface pH of 7.34, which is approximately 0.4 pH units higher than the steady‐state surface pH in colonoids from the transverse colon. This regional variation recapitulates in vivo wireless capsule data showing higher luminal pH in the distal versus proximal colon in healthy individuals [7], and aligns with prior observations of differential alkalinization rates across murine intestinal segments, with particularly high rates in the mid‐distal colon that correlate with DRA expression levels [11, 21].

In the proliferative organoids with minimal baseline DRA expression, both genetic CFTR deficiency (CF organoids) and pharmacological inhibition of CFTR significantly reduced surface pH, confirming the role of CFTR as a bicarbonate transporter. DRA deletion also reduced surface alkalinization, which was surprising given the low DRA expression levels in the nondifferentiated colonic organoids. DRA overexpression not only enhanced initial alkalinization rates but completely restored steady‐state surface pH in both CF organoids and CFTR‐inhibited colonoids. The initial alkalinization rates in HL and CF organoids with DRA overexpression did not differ, nor did the steady‐state surface pH of DRA_OE HL and DRA_OE CF organoids. The heterologously expressed DRA was found in small subapical vesicles and in the plasma membrane of both DRA_OE HL and DRA_OE CF vesicles (Figure 5), similar to the localisation of endogenous DRA in differentiated organoids [40], The data suggest that DRA functions as the dominant regulator of surface pH homeostasis and its function and membrane localization are independent of a functional CFTR. We did not study stimulation‐associated DRA trafficking in detail, however. The effect of DRA on surface pH regulation is consistent with previous findings in mouse models [8, 12], showing more pronounced pH microclimate acidification in *slc26a3* ^−/−^ mice compared to *cftr* ^−/−^ mice. Interestingly, DRA overexpression is able to establish and maintain an alkaline surface pH in the absence of functional CFTR as well as NHE3, since NHE3 mRNA expression is minimal in nondifferentiated, proliferative organoids [40]. This suggests that DRA transport itself responds to shifts in ion gradients. The ability of DRA to compensate for CFTR dysfunction supports recent findings demonstrating that linaclotide can stimulate bicarbonate secretion through enhanced DRA membrane localization even during loss of CFTR activity [33].

The forskolin‐stimulated alkalinization responses provided additional insights. DRA‐overexpressing cultures achieved maximal alkalinization more rapidly than controls. CFTR‐compromised cultures showed gradual, attenuated responses. However, when DRA expression was induced in CF rectal organoids, surface pH after forskolin was not different between HL and CF organoids. Clearly, the lack of a functional CFTR does not fully abrogate the increase in surface pH after forskolin. Several potential mechanisms are feasible: Since the basolateral bath contains ${\mathrm{HCO}}_{3}^{–}$, an increase in paracellular permeability for anions is one potential CFTR independent mechanism. Effects of second messengers on the pore selectivity and permeability of tight junctional complexes are feasible, and have been described [55, 56, 57]. Another potential mechanism is a cAMP‐mediated inhibition of proton exporters such as NHE3 and the colonic H^+^/K^+^ ATPase [13, 58, 59]. Although the low expression of both proton exporters in the proliferative, nondifferentiated organoids makes this effect less likely, we also observed a decrease in surface pH in the DRA‐deleted colonoids compared to HL controls, despite low endogenous DRA expression in the latter.

CFTR function in nondifferentiated intestinal organoids is often assessed by measuring the increase in organoid diameter during forskolin‐induced swelling [60]. While DRA overexpression corrected the abnormal eccentric morphology of CF organoids and enhanced initial swelling rates in HL organoids during forskolin stimulation, these effects were modest. However, DRA overexpression could not restore swelling in CF organoids or in HL organoids after effective CFTR inhibition. These observations are consistent with recent findings demonstrating that neither DRA‐OE, knockdown, nor pharmacological inhibition of DRA significantly influences forskolin‐induced swelling responses in human colonic/rectal enteroids [50]. Electrophysiological assessment of electrogenic anion efflux in Ussing chambers is another method of assessing CFTR function [61]. Ussing chamber experiments demonstrated that DRA overexpression did not enhance CFTR‐mediated electrogenic anion secretion in HL rectal organoids. The equivalent short‐circuit current responses to forskolin were indistinguishable between vehicle controls and DRA‐overexpressing monolayers.

This suggests to us that in native enterocytes from the cryptal regions of the intestine, which display high expression levels for NKCC1, AE2 and NBCe1/n1 [40], a heterologous expression of DRA results in enhanced apical ${\mathrm{HCO}}_{3}^{–}$ output, but does not influence CFTR‐mediated anion efflux. This contrasts with the previously reported robust CFTR activation by DRA reported in Xenopus oocytes [28]. One reason for the discrepancy may be that in the low or absent expression of endogenous anion uptake pathway, the activation of Cl^−^ efflux via heterologously expressed CFTR may induce SLC26A3‐mediated Cl^−^ uptake. In intestinal epithelium, however, basolateral anion uptake mechanisms (NKCC1, AE2) maintain intracellular chloride availability [62, 63]. It has been demonstrated that in the shark rectal gland, which apically expresses CFTR, the rate‐limiting factor for Cl^−^ secretion is basolateral Cl^−^ uptake [64]. Likewise, apical CFTR‐dependent ${\mathrm{HCO}}_{3}^{–}$ secretion depends on the expression of basolateral anion uptake transporters and intracellular ${\mathrm{HCO}}_{3}^{–}$ generation [65, 66, 67, 68]. Elegantly shown both experimentally and mathematically, the high ${\mathrm{HCO}}_{3}^{–}$ concentration in pancreatic juice of humans and guinea pigs is due to a low expression of basolateral Cl^−^ uptake mechanisms, which results in very low intracellular Cl^−^ concentrations upon CFTR stimulation and increases ${\mathrm{HCO}}_{3}^{–}$ permeability of the CFTR channel [69, 70]. In contrast, both the CFTR high expresser cells in the villous region of the small intestine [71], and the cells in the small intestinal and colonic crypts and base of the villi, express NKCC1, NBCs and AE2 [72, 73, 74], as do the human nondifferentiated organoids [40]. In contrast, both the nondifferentiated cells in the cryptal region of the small intestine and colon as well as the Best4 positive CFTR high expresser cells in the small intestine do not express DRA [43, 71]. Recent studies in human small intestinal organoids suggested that organoids consisting of the differentiated villous cells only secrete fluid into their lumen if they express the Best4 positive CFTR high expresser cells, which do not express DRA [75]. Thus, colocalization of CFTR and DRA in the human intestine may be limited to the cells in the transitioning zone between the transient amplifying cells and the mature absorptive enterocytes. In contrast, the murine small intestine does not express CFTR high expressers cells and may express CFTR at low levels more diffusely in the villous epithelium (while the highest expression is found in the crypts) [76]. Nevertheless, the absence of DRA did not significantly reduce the linaclotide‐induced Isc response in murine jejunum, while it reduced the ${\mathrm{HCO}}_{3}^{–}$ secretory response by 30% [77]. Similar results were obtained during cAMP‐dependent stimulation of bicarbonate secretion in mouse duodenum [78]. However, this agonist‐induced DRA dependent increase in ${\mathrm{HCO}}_{3}^{–}$ secretion is in part due to agonist‐mediated NHE3 inhibition, and is also seen in the complete absence of CFTR expression [8, 13].

In conclusion, the heterologous expression of DRA did not result in a significant enhancement of anion and fluid flux, which was fully CFTR‐dependent. Vice versa, the function of DRA as a base exporter was not influenced by the presence of a functional or a nonfunctional CFTR, which was not present in the plasma membrane [39]. The largely independent function of the two anion transporters in the colon confirms results from studies in the mouse colon, where the genetic deletion of CFTR did not reduce basal alkalization rates in isolated mid‐distal colonic mucosa and only minimally reduced the forskolin‐induced alkalization rates that were not due to NHE3 inhibition [79]. In contrast, the basal as well as FSK‐induced alkalization rates were very low in the mid‐distal colon of the DRA‐deleted mouse, while FSK‐induced Isc was almost identical to that in the WT [8]. In addition, colonic manifestations differ substantially between DRA and CFTR deficiency in both mice and patients [80, 81, 82, 83].

Beyond its role in surface pH, DRA expression profoundly influenced intracellular mucin distribution in CF organoids. The altered mucin processing in association with perturbed mucosal bicarbonate secretion is well documented. At the luminal surface of the colon, deficiencies in either DRA or CFTR are associated with abnormalities in the structure and thickness of the mucus layer [8, 12, 52]. Additionally, at the cellular level, goblet cell hyperplasia and defective mucin degranulation are characteristic features of CF pathology in both mouse intestine [10] and human patients [84]. Investigation of goblet cell mucin exocytosis dynamics in mouse intestinal tissue has revealed incomplete mucin secretion by goblet cells in response to carbachol in CF intestine, contrary to wild‐type tissue. Importantly, this phenotype is independent of the inflammatory and/or CF intestinal environment, as it was reproducible in enteroid cultures [10]. In our experiments, intracellular mucin distribution was assessed in fixed rectal organoids. Unlike healthy organoids, CF organoids displayed altered mucin distribution patterns in goblet cells characterized by larger, more densely packed mucin granules with reduced abundance in the subapical compartment. Expression of DRA normalized this aberrant pattern and improved intracellular mucin distribution. The molecular mechanism behind these observations demands further investigation; however, it likely involves pH‐dependent mucin packaging and secretion. Mucin packing within goblet cell secretory granules has been demonstrated to be pH and calcium‐dependent [85]. Furthermore, mucin granules in goblet cells of CF enteroid cultures maintain an abnormally alkaline pH compared to wild‐type cultures [10], and this pH imbalance may have been corrected upon restoration of bicarbonate efflux through the induction of DRA in CF rectal organoids in our experiments. In the cryptal mouth cells of murine colonic crypts, the intracellular pH was significantly more alkaline in *slc26a3* ^−/−^ compared to WT colonocytes, demonstrating the important function of DRA as a base extruder in colonocytes [12].

Our study has both advantages and limitations. Advantages are the inducible expression of DRA in a well‐characterized organoid system which expresses endogenous CFTR as well as the physiological anion uptake mechanisms, preventing a “nonphysiological” coupling of CFTR‐dependent Cl^−^ efflux to DRA as the predominant Cl^−^ uptake mechanism. The Tet‐on system for DRA expression prevents differences in the cellular characteristics prior to the DRA induction and ensures comparability between the DRA‐OE and respective control organoids. The ratiometric surface pH measurements allow assessment of DRA‐ and CFTR‐dependent and independent pH‐, HCO_3_‐ and agonist‐induced alkalinization, as well as steady‐state surface pH measurements.

One limitation is the fact that our organoids are still a gene manipulated model system, expressing both endogenous DRA (in low amounts, in the nondifferentiated state of the organoids we use) and a flag‐tagged human DRA. Although we inserted the tag to the N‐terminus, which does not contain the PDZ‐motif and the CFTR interaction site, and found the heterologous DRA in the brush border membrane and subapical vesicles similar to the native DRA in differentiated organoids, we do not exactly know the consequences. In addition, expressing DRA in nondifferentiated organoids has the advantage of ample CFTR in the brush border membrane, but there is of course the caveat that DRA may not find all the necessary interaction partners, some of which we do not know yet. Another limitation is that we have not studied the potential importance of DRA as an apical Cl^−^ uptake mechanism for the very tiny residual CFTR‐dependent cAMP‐mediated anion efflux in the differentiated organoid monolayers [39]. Although we stably deleted DRA expression in the human organoids by CRISPR/Cas9 and these organoid monolayer showed secretagogue‐induced changes in the potential difference that were identical to those in the controls, their TEER was lower than in the controls at the same time on the filters, and subtle changes in the expression levels of several genes were observed. This would render the results difficult to explain, given the very low CFTR activity in the differentiated organoids.

In summary, our study demonstrates that both CFTR and more so the SLC26A3 (DRA) anion exchanger independently alkalize the colonic surface pH. In human colonic organoids with either high endogenous expression of functional CFTR, or of a nonfunctional F508mutant protein, the induction of heterologous DRA expression resulted in equally high surface alkalization rates. Conversely, the anion and fluid secretory activity of CFTR was not significantly influenced by induction of DRA expression. Moreover, DRA expression was unable to rescue the fluid secretory defect in F508 mutant organoids. However, DRA expression normalized the high eccentricity and the abnormal mucus granule distribution in F508 mutant organoids, possibly via its effect as a major base extruder and pH regulator in the colonocytes. In conclusion, DRA rescues some but not all of the abnormal cellular physiology in CF mutant colonocytes. DRA and CFTR anion transport functions predominantly independent of each other in the native colonocyte.

## 4 Material and Methods

### 4.1 Human Subjects

Human biopsies used for establishing intestinal organoid cultures were in part previously reported by our group [39]. All biopsies were obtained with informed written consent of the patients, in accordance with the guidelines of the Declaration of Helsinki and approved by the Institutional Review Board of Hannover Medical School (Nr. 8535_BO_K_2019, Nr. 8922_BO_S_2020, Nr. 11361_BO_K_2024).

### 4.2 Human Intestinal Organoid Cultures

The establishment of 3D and monolayer organoid cultures from intestinal biopsies has been previously reported [39]. Organoid lines were established from the transverse colon of a healthy donor (HL1, 35‐year‐old male), the rectum of another healthy donor (HL2, 19‐year‐old female), and the rectum of a donor with cystic fibrosis (CF17, 19‐year‐old male, F508del/F508del) for detailed functional analyses. For validation studies, additional organoid cultures from healthy donors (HL3, 61‐year‐old female and HL243, 26‐year‐old female) and CF donors (CF3, 17‐year‐old female, F508del/N1303K and CF22, 41‐year‐old female, F508del/F508del) were employed (Table S1) [39]. The culture media composition is detailed in Table 1. For routine maintenance of 3D cultures, organoids were initially treated with stemness medium for 2 days following passage, then cultured in expansion medium for 3–4 days. For functional analyses, monolayer cultures were established on Transwell inserts with polyester membranes (Corning 3470 and 3472). Monolayers followed the same treatment protocol (stemness medium for 2 days, then expansion medium for 3–4 days) before experimental use. Monolayer integrity was confirmed by transepithelial electrical resistance (TEER) measurements exceeding 300 Ω cm^−2^ using an EVOM2 device (World Precision Instruments, Sarasota, FL, USA).

**TABLE 1:** Composition of different organoid culture media.

Reagent	Source	Final conc.	Stemness medium	Expansion medium
L‐WRN conditioned medium	According to ref. [86]	50% v/v	✓	✓
Advanced DMEM/F12	Invitrogen, 12 634–028	45% v/v	✓	✓
HEPES 1 M	Invitrogen, 15 630–056	10 mM	✓	✓
GlutaMAX‐I	Invitrogen, 35 050–079	2 mM	✓	✓
Primocin	Invivogen, ant‐pm‐1	100 μg/mL	✓	✓
N2 supplement	Invitrogen, 17 502–048	1×	✓	✓
B27 supplement	Invitrogen, 17 504–044	1×	✓	✓
N‐Acetylcysteine	Sigma‐Aldrich, A9165	1 mM	✓	✓
Human recombinant EGF	Invitrogen, PMG8043	50 ng/mL	✓	✓
A‐83‐01	Tocris, 2939	500 nM	✓	✓
SB202190	Tocris, 1264	10 μM	✓	✓
Nicotinamide	Sigma‐Aldrich, N0636	10 mM	✓	✓
[Leu15]‐Gastrin I	Sigma‐Aldrich, G9145	10 nM	✓	✓
CHIR99021	Sigma‐Aldrich, SML1046	10 μM	✓	—
Y‐27632 dihydrochloride	Tocris, 1254	10 μM	✓	—

To enable inducible DRA expression, the human SLC26A3 coding sequence was PCR‐amplified and tagged with 3xFlag at the N‐terminus, then cloned into the pLIX_403 backbone (a gift from David Root (Addgene plasmid # 41395; http://n2t.net/addgene:41395; RRID:Addgene_41 395)) under a Tet‐On promoter. Lentiviral particles were produced from this construct and an empty backbone control (vehicle) in HEK293T cells, then used to transduce organoid cultures. Puromycin‐resistant stable clones were isolated and maintained under standard culture conditions. For induction experiments, doxycycline (5 μM final concentration) was added to both 3F‐DRA and vehicle control cultures 48 h prior to analysis.

For generation of DRA‐knockout (DRA‐KO) colonoid cultures, a single guide RNA (sgRNA) targeting the SLC26A3 gene (sequence: GGGATTGTGGCCGTACTACA) was designed using CRISPick (RRID:SCR_025148, Broad Institute, https://portals.broadinstitute.org/gppx/crispick/public). The sgRNA was cloned into lentiCRISPR v2 (a gift from Feng Zhang; Addgene plasmid #52961; http://n2t.net/addgene:52961; RRID:Addgene_52 961 [87]) and used to produce lentiviral particles in HEK293T cells. Human colonoids (HL1 donor) were transduced with these lentiviral particles and selected with 2 μg/mL puromycin. Initial evaluation of the targeted region by PCR amplification and Sanger sequencing revealed a 91% indel rate, as determined using the ICE CRISPR Analysis tool by EditCo Bio (https://ice.editco.bio/#/). Subsequently, single‐cell colonies were generated from the transduced population, propagated, and validated for DRA knockout by Sanger sequencing and western blot analysis.

### 4.3 Apical Surface Fluid pH Measurement

We assessed the surface pH of intestinal monolayer cultures using a modified semi‐automated fluorescent plate reader assay [22] developed based on the method established by Saint‐Criq et al. [42]. Three distinct apical solutions were prepared and sterile filtered: bicarbonate containing Krebs buffer solution (KRB, in mM: 25 NaHCO_3_, 115 NaCl, 5 KCl, 1 CaCl_2_, 1 MgCl_2_), Tris–NaCl solution (8.6 mM Tris base, 150 mM NaCl), and 154 mM NaCl solution. Equilibration of unbuffered NaCl solution at 37°C and 5% CO_2_ yields a acidic solution (pH ~5), which was titrated to pH 7 using 8.6 mM Tris base. The buffer capacity of 8.6 mM Tris is 14.3 times lower than that of the physiological ${\mathrm{HCO}}_{3}^{–}$/CO_2_ system and therefore, even if persisting during the assay, considered negligible in comparison, allowing for detection of endogenous bicarbonate buffering capacity without significant interference from the Tris buffer system (Figure S1).

On the day of experiments, culture media were refreshed and incubated for 2 h. The inserts were washed with the designated apical solution on the apical side and KRB on the basolateral side. The inserts were then transferred to a new 24‐well plate containing 400 μL KRB with glucose (in mM: 25 NaHCO_3_, 115 NaCl, 5 KCl, 1 CaCl_2_, 1 MgCl_2_, 5 glucose) in the inner wells, pre‐equilibrated in the cell culture incubator. To maintain optimal humidity, we did not use the outer wells and filled them with 1 mL sterile and deionized water. Measurements were performed using a Spark fluorescent plate reader (Tecan, Crailsheim, Germany) at 37°C and 5% CO_2_, with a large humidity cassette filled with 6 mL deionized water at each side and prewarmed in the plate reader. The background fluorescence for the inserts was measured using excitation filters at 485/20 and 535/25 nm, and emission filters at 535/25 and 595/35 nm for pH‐insensitive and pH‐sensitive intensities, respectively. Afterwards, the apical surface received 2 μg each of Alexa Fluor 488‐dextran and pHrodo Red‐dextran dyes (Thermo Scientific, Darmstadt, Germany) in a total of 10 μL of the respective apical solution. Real‐time measurements were recorded at 10‐min intervals, with 5 readings per sample at each time point for proper coverage of the insert area. After establishing steady‐state surface pH, we added forskolin to the basolateral chamber from a 25× stock solution in KRB to reach the final concentration 10 μM. Where specified, apical and/or basolateral solutions were supplemented with 5 μM doxycycline or 10 μM CFTR_inh_172. Of note, we identified that after inserting the plate into the device, approximately 15 min for initial temperature and CO_2_ equilibration is needed. Therefore, readouts within this timeframe, both at the beginning of the experiment and after forskolin addition, were excluded to ensure reliable measurement. For pH calibration, we performed two in situ steps at pH 8 and pH 6, each lasting minimum 1 h without CO_2_. The calibration solutions contained 86 mM NaCl, 5 mM KCl, 1.2 mM CaCl_2_, 1.2 mM MgCl_2_, with either 100 mM Tris (pH 8) or 100 mM MES (pH 6). For pH 8 calibration, after removing HCO_3_‐KRB from the basolateral chamber, we added 50 μL calibration buffer to the apical and 400 μL to the basolateral chamber. The addition of 50 μL pH 8 calibration buffer to the existing apical solution containing dyes maintained pH stability due to its strong buffering capacity, and we confirmed that the fluorescence ratio of the dyes at pH 8 showed no significant difference between 10 and 60 μL total apical solution volumes. For pH 6 calibration, apical and basolateral chambers were washed with the calibration buffer and then aspirated. 10 μL of pH 6 calibration containing 2 μg of each dye as above was carefully added to the apical chamber and 400 μL of the buffer was added to the basolateral chamber. Data analysis involved subtracting mean background from mean value for each data point at each wavelength, followed by calculation of the ratio between pH‐sensitive and pH‐insensitive fluorescence. These fluorescent ratios were then converted to pH values using individual calibration curves for each sample.

### 4.4 Electrophysiological Measurements of Ion Transport

Ussing chamber experiments were conducted using organoid monolayer cultures according to previously established protocols [39]. Cultures were treated with a final concentration of 5 μM doxycycline for 2 days prior to experiment. Pharmacological agents were applied at the following final concentrations: amiloride (10 μM, luminal side), forskolin (sequentially 1.25, 5 and 10 μM, serosal side), IBMX (100 μM, serosal side), UTP (100 μM, luminal side), and bumetanide (100 μM, serosal side). Equivalent short‐circuit current (*I*_eq_) was calculated using Ohm's law, where *I*_eq_ = *V*_te_/*R*_te_. To determine Δ*I*_eq_, the average peak response following drug application was subtracted from the baseline average measured during the 2‐min interval prior to drug administration.

### 4.5 Immunofluorescence and Confocal Microscopy

Whole‐mount 3D organoid cultures were prepared for immunofluorescence imaging following a previously published protocol [88]. Primary antibodies were used against SLC26A3 (1:100, sc‐376 187, Santa Cruz Biotechnology) and MUC2 (1:500, ab272692, Abcam, Cambridge, UK). Detection was performed using AlexaFluor‐conjugated secondary antibodies: Goat anti‐mouse 488 (Cat. No. A11029) and Goat anti‐rabbit 568 (Cat. No. A11011) from Invitrogen. Nuclei were counterstained with DAPI, and F‐actin was visualized using Phalloidin‐iFluor647 Reagent. For alternative mucus visualization, *Ulex europaeus* agglutinin‐1 (UEA1) lectin coupled to Rhodamine dye (Vector Laboratories) was employed [89]. Images were acquired using a TCS SP8 confocal microscope (Leica Microsystems) with either a 20× multi‐immersion or 63× oil immersion objective. Subsequent image processing and analysis were performed using LAS X (Leica Microsystems), Imaris (version 8, Oxford Instruments), and Fiji software. For quantitative analysis of MUC2 distribution, the intensity profile along the longitudinal axis of individual goblet cells was measured using the intensity profiler tool in Fiji. Signal intensities were normalized to total fluorescence intensity, and positional data was converted to percentage of total cell length, with 0% representing the basolateral membrane and 100% the apical surface. This approach enabled standardized comparison of MUC2 distribution patterns among different cells and different models. Statistical dispersion in MUC2 intracellular distribution among different samples was assessed by calculating the interquartile range, which represents the range between the 25th and 75th percentiles of the MUC2 signal.

### 4.6 Organoid Morphometric and Forskolin‐Induced Swelling Assays

Morphometric analysis of 3D intestinal organoid cultures was performed using bright‐field imaging with a Zeiss Axio light microscope. Images were captured at 10× magnification and analyzed for eccentricity using OrganoSeg software according to the method described by Borten et al. [90].

Forskolin‐induced swelling assay (FIS) was conducted according to the published protocol by Vonk et al. [91] using 10 μM final concentration of forskolin. Where applicable, cultures were pre‐incubated with 10 or 50 μM CFTR_inh_172 or corresponding concentration of DMSO (0.1% v/v) as vehicle control, 2 h prior to the experiment. These concentrations were maintained in the forskolin‐containing medium added to the samples immediately before starting the experiment. Fluorescence images were obtained using a Zeiss LSM 980 microscope with a 5× objective at 37°C and 5% CO_2_. Image processing and analysis were performed using ZEN 2.5 (blue edition), CellProfiler (V4.2.8) and R (V4.4.2) softwares according to the open‐source workflow published by Hagemeijer et al. [92].

### 4.7 Western Blot

SDS‐PAGE and Western blot were performed as reported previously [40]. Antibodies against SLC26A3 (1:500, Santa Cruz Biotechnology), beta‐actin (1:2000, Cat. No. 3700, Cell Signaling Technology, Leiden, Netherlands) and flag‐epitope (1:5000, Cat. No. F3165, Sigma‐Aldrich) as well as Horseradish Peroxidase‐conjugated secondary antibodies (1:10,000, Cat. No. G‐21040 or G‐21234, Invitrogen, Karlsruhe, Germany) were used for immunoblotting. The signals were visualized by Enhanced chemiluminescence solution (Cat. No. RPN2209, Cytiva) and analyzed by Fusion FX system (Vilber Lourmat, Eberhardzell, Germany).

### 4.8 Data Analysis and Statistics

Detailed functional analyses were conducted using multiple passages from one biological sample per condition, i.e., healthy colonoids from HL1, healthy rectal organoids from HL2, and CF F508del rectal organoids from CF17. Validation studies employed additional organoid lines (healthy: HL3, HL243; CF: CF3, CF22) as biological replicates for selected experiments. Technical replicates represent independent measurements or organoid cultures from the same donor line. Unless otherwise specified, *n* values in figures and figure legends represent technical replicates, with the donor line(s) indicated in each figure legend.

Data analysis and visualization were performed using Microsoft Excel 2016 and GraphPad Prism (version 8.0.2, GraphPad Software Inc., San Diego, CA, USA) software. Results were expressed as mean ± SEM. For experiments comparing more than two groups, one‐way ANOVA was performed followed by Tukey's post hoc test to correct for multiple comparisons. For pairwise comparisons, Student's *t*‐test was used. Statistical significance levels were denoted as: ns (not significant) *p* > 0.05, *p ≤ 0.05, **p ≤ 0.01, or ***p ≤ 0.001. An artificial intelligence language model (Claude, Anthropic, San Francisco, CA, USA) was occasionally utilized to check and refine the language presentation of this manuscript. All intellectual content remains the original work of the authors.

## Author Contributions

Mahdi Amiri, Ph.D.: (conceptualization: lead; methodology: lead; validation: equal; formal analysis: lead; investigation: lead; resources: supporting; data curation: equal; writing – original draft: lead; writing – review and editing: equal; visualization: lead; project administration: equal; funding acquisition: supporting). Azam Salari, Dr. rer. nat. (conceptualization: supporting; methodology: equal; validation: equal; formal analysis: equal; investigation: lead; data curation: equal; writing – original draft: supporting; writing – review and editing: equal; visualization: equal). Ursula Seidler, Prof. Dr. (conceptualization: lead; validation: supporting; resources: lead; writing – original draft: lead; writing – review and editing: equal; project administration: lead; funding acquisition: lead).

## Funding

This study was supported by the Deutsche Forschungsgemeinschaft (DFG) through grants Se 460/19‐1 and Se 460/22‐1 to U.S. and AM 786/1‐2 to M.A., and by the Volkswagen Stiftung through grant Z1953 to U.S.

## Ethics Statement

Human biopsies were obtained with informed consent of the patients, in accordance with the guidelines of the Declaration of Helsinki, and approved by the Institutional Review Board of Hannover Medical School (Nr. 8535_BO_K_2019, Nr. 8922_BO_S_2020, Nr. 11361_BO_K_2024).

## Conflicts of Interest

The authors declare no conflicts of interest.

## Supporting information


**Data S1:** Supplementary Figures and Table.

## Data Availability

The data that support the findings of this study are available from the corresponding author upon reasonable request.
