# Supplementary material for: Distinct Roles of SLC26A3 and CFTR in Surface pH Regulation and Bicarbonate Secretion in Human Intestinal Epithelium
Source: Acta Physiol (Oxf). 2026 Jan 7;242(2):e70157. doi: 10.1111/apha.70157 (PMC12777511; doi:10.1111/apha.70157)
Supplement: Supplementary file 1 — Data S1: Supplementary Figures and Table. [file APHA-242-e70157-s001.pdf]

Supplementary Table

Table S1: Donor characteristics and biopsy locations for human intestinal organoid lines used in this study.

Sample	Age (years)	Gender	Biopsy sections	CFTR Genotype
**HL1**	35	Male	Transverse Colon	Healthy
**HL2**	19	Female	Rectum	Healthy
**HL3**	61	Female	Colon and rectum	Healthy
**HL243**	26	Female	Colon and rectum	Healthy
**CF3**	17	Female	Rectum	F508del/N1303K
**CF17**	19	Male	Rectum	F508del/ F508del
**CF22**	41	Female	Rectum	F508del/ F508del

Supplementary Figures

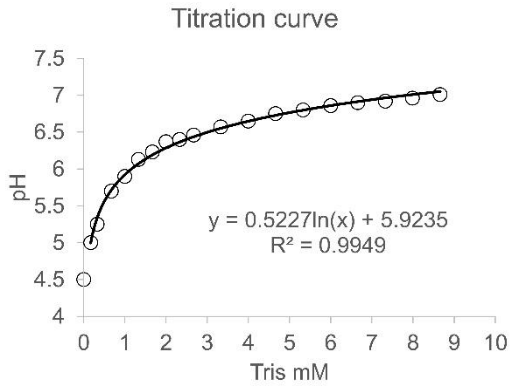

Figure S1: Titration curve of Tris in the presence of 5% CO_2. By titrating carbogen-gassed (5% CO_2/95% O_2; PCO_2= 40mmHg) distilled water at 37°C with increasing concentrations of Tris buffer, we determined that addition of 8.6mM Tris is sufficient to neutralized the initial acidification of water.

Under these conditions, buffer capacities were calculated using the Van Slyke equation

$\beta = 2.303 \times C \times \alpha \times (1\text{-}\alpha)$, where C = total buffer concentration ([HA] + [A^-]), α = fraction in deprotonated form = Ka/(Ka + [H^+]), Ka = acid dissociation constant at 37°C, [H^+] = $10^{-7.4}$ = 3.98×10^{-8} M. Accordingly:

i) The buffer capacity (β) of the 8.6 mM Tris buffer system is calculated as:

$\alpha = Ka/(Ka + [H^+]) = (1.58 \times 10^{-8})/(1.58 \times 10^{-8} + 3.98 \times 10^{-8}) = 0.285$

$\beta_{Tris} = 2.303 \times (8.6 \times 10^{-3}) \times 0.285 \times (1 - 0.285) = 4.03$ mM/pH unit

ii) The buffer capacity (β) of HCO_3^-/CO_2 system with 25mM steady state bicarbonate concentration, where infinite CO_2 reservoir at experimental conditions eliminates dependence on the $\alpha \times (1\text{-}\alpha)$, is calculated as below:

$\beta_{HCO_3^-} = 2.303 \times [HCO_3^-] = 57.6$ mM/pH unit

This confirms that even if the initial Tris concentration is maintained after reaching steady state, the buffer capacity of 8.6 mM Tris is 14.3 times lower than that of the physiological HCO_3^-/CO_2 system. Tris accounts for only 6.5% of the total buffering capacity, leading to a minor 6.5% reduction in pH sensitivity to bicarbonate transport. This minimal interference supports the use of Tris for initial pH adjustment without significantly compromising the accuracy of bicarbonate transport measurements.

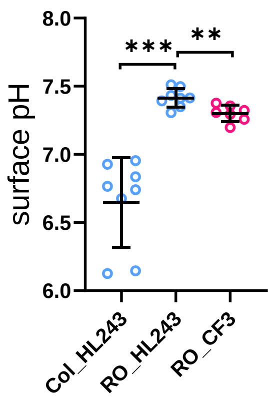

Figure S2: Surface pH measurements in organoid cultures from additional donors confirm model-specific differences in steady-state pH profiles. Colonoids and rectal organoids from healthy donor

HL243 and rectal organoids from CF donor CF3 replicate the distinct pH responses observed between culture systems. Data represent mean ± SEM. **P < 0.01, ***P < 0.001.

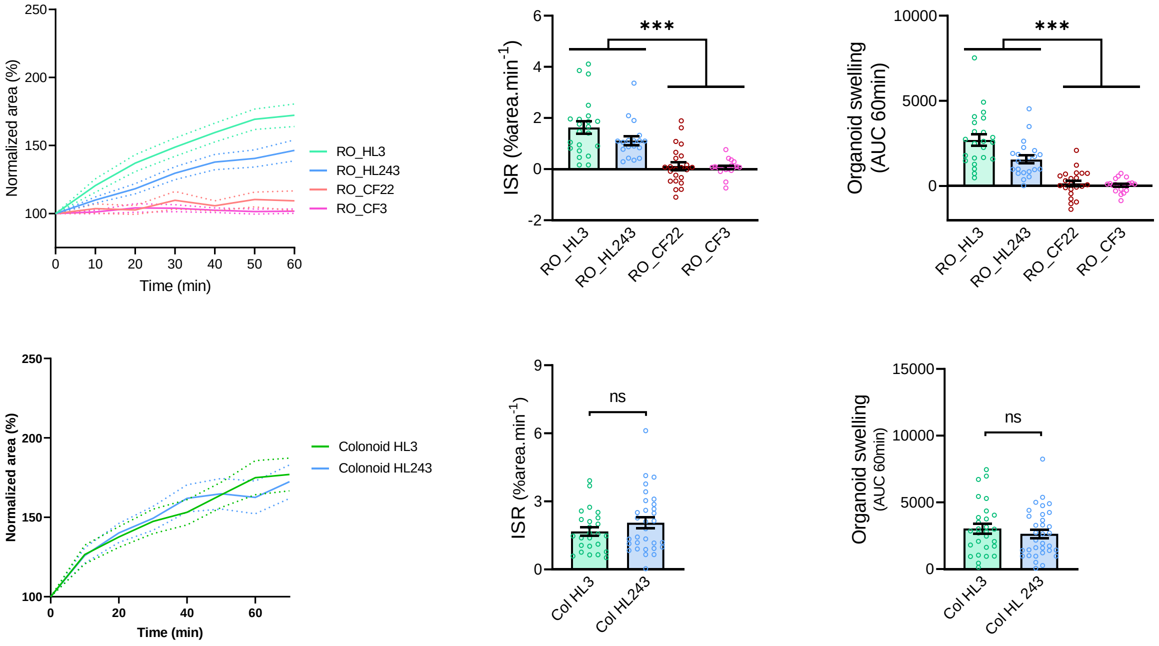

Figure S3: Forskolin-induced swelling assay in organoid cultures from additional donors confirms model-specific CFTR-mediated fluid secretion responses. Time-course analysis of normalized area expansion and quantitative measurements of initial swelling rate (ISR) and cumulative organoid swelling (AUC) in rectal organoids from healthy donors (RO_HL3, RO_HL243) and CF donors (RO_CF22, RO_CF3) (top panels), and colonoids from healthy donors (Col_HL3, Col_HL243) (bottom panels). Rectal organoids from CF donors demonstrate complete absence of forskolin responsiveness with abolished ISR and swelling parameters compared to healthy controls. Colonoids from both healthy donors show robust and comparable swelling responses. These findings validate the distinct fluid secretion capacities between model systems and confirm the complete loss of CFTR-mediated swelling in CF rectal organoids across multiple donor. Mean ± SEM; two-tailed parametric Student's t-test or ANOVA as appropriate ***P < 0.001, ns = not significant.
